# Baseline-dependent effect of dopamine’s precursor L-tyrosine on working memory gating but not updating

**DOI:** 10.3758/s13415-020-00783-8

**Published:** 2020-03-04

**Authors:** Bryant J. Jongkees

**Affiliations:** 1 Institute of Psychology & Leiden Institute for Brain and Cognition, Leiden University, Wassenaarseweg 52, 2333AK Leiden, the Netherlands; 2 Princeton Neuroscience Institute, Princeton University, Princeton, NJ USA

**Keywords:** Dopamine, L-tyrosine, Working memory, Updating, Gating, Individual differences

## Abstract

Adaptive goal-directed behavior requires a dynamic balance between maintenance and updating within working memory (WM). This balance is controlled by an input-gating mechanism implemented by dopamine in the basal ganglia. Given that dopaminergic manipulations can modulate performance on WM-related tasks, it is important to gain mechanistic insight into whether such manipulations differentially affect updating (i.e., encoding and removal) and the closely-related gate opening/closing processes that respectively enable/prevent updating. To clarify this issue, 2.0 g of dopamine’s precursor L-tyrosine was administered to healthy young adults (*N* = 45) in a double-blind, placebo-controlled, within-subjects study. WM processes were empirically distinguished using the reference-back paradigm, which isolates performance related to updating, gate opening, and gate closing. L-tyrosine had a selective, baseline-dependent effect only on gate opening, which was evidenced by markedly reduced variance across subjects in gate opening performance in the L-tyrosine compared with the placebo condition, whereas the whole-sample average performance did not differ between conditions. This indicates a pattern of results whereby low-performing subjects improved, whereas high-performing subjects were impaired on L-tyrosine. Importantly, this inverted U-shaped pattern was not explained by regression to the mean. These results are consistent with an inverted-U relationship between dopamine and WM, and they indicate that updating and gating are differentially affected by a dopaminergic manipulation. This highlights the importance of distinguishing these processes when studying WM, for example, in the context of WM deficits in disorders with a dopaminergic pathophysiology.

**Electronic supplementary material:**

The online version of this article (10.3758/s13415-020-00783-8) contains supplementary material, which is available to authorized users.

## Introduction

Adaptive goal-directed behavior relies on efficient working memory (WM), the process whereby the brain maintains a limited amount of information in service of present task-goals (Baddeley, 1992; Baddeley & Hitch, 1974). WM involves both the ability to select and maintain task-relevant information and to remove/update this information as subgoals and circumstances change (Hazy, Frank & O’Reilly, 2006; Rac-Lubashevsky & Kessler, 2016a). According to prominent neurocomputational models (Chatham & Badre, 2015; Frank, Loughry, & O’Reilly, 2001; Hazy et al., 2006; O’Reilly, 2006), the balance between the functionally opposite states of robust maintenance and flexible updating is controlled by an input-gating mechanism, which dynamically regulates the access of input to WM. The gate is “closed” when neural gain in prefrontal cortex (PFC) is high; in this state, implemented by tonic inhibition of the thalamus and sufficiently high tonic dopamine levels in PFC (Durstewitz & Seamans, 2008), there is strong recurrent excitation within and mutual inhibition between neuronal populations. This facilitates the maintenance of representations and prevents intrusion of WM by new input. However, phasic dopamine release in basal ganglia (BG) can trigger the gate to “open,” i.e., there is a momentary disinhibition of the thalamus and a decrease in neural gain within PFC (Hazy et al., 2006). In this state, the transiently reduced inhibition between neuronal populations serves to facilitate updating of WM content.

Given the strong dopaminergic involvement in WM, there has been great interest in enhancing WM function by manipulating the dopaminergic system. Studies have, for example, shown improved performance on WM-related tasks following administration of dopamine’s precursors L-tyrosine (Jongkees, Hommel, Kühn, & Colzato, 2015; Jongkees et al., 2017) and L-dopa (Alavash et al., 2018; Eckart, Fuentemilla, Bauch, & Bunzeck, 2014), as well as the dopamine D2 receptor agonist bromocriptine (Gibbs & D’Esposito, 2005b; Wallace, Vytlacil, Nomura, Gibbs, & D’Esposito, 2011). However, there often is a lack of mechanistic insight into the neurocognitive basis of these effects; it frequently remains unclear which specific components of WM are affected by the dopaminergic manipulation. Although in previous research efforts have been made to disentangle several components (Gibbs & D’Esposito, 2005a), there often is no distinction between the efficiency of several closely related but separate processes: (1) updating (involving removal of old and encoding of new WM content) versus; (2) the gate opening process, which presumably precedes and enables—but does not constitute—updating versus; (3) the gate closing process, which prevents irrelevant input from being gated into WM (Rac-Lubashevsky & Kessler, 2016a).

This matter is important to clarify, because theoretical accounts suggest that dopamine activity in PFC versus BG modulate different components of WM, such as updating/maintenance versus gating, respectively (Braver & Cohen, 2000; Durstewitz & Seamans, 2008; Hazy et al., 2006). Dopaminergic manipulations that particularly target one neural network or the other therefore could have different and perhaps even opposite cognitive-behavioral effects. Furthermore, neuropsychiatric disorders related to dopaminergic pathophysiology may involve specific deficits in some but not other component processes. For example, WM deficits in patients with major depression are attributed to dysfunctional updating of negative content (Zetsche, Bürkner, & Schulze, 2018), but it is possible that this relates either to a prefrontal deficit in clearing WM or instead a BG deficit in control over input-gating of negative information. This points to a strong need for carefully disentangling component processes of WM when studying the effects of dopaminergic manipulations or dysfunctional WM in neuropsychiatric disorders. Therefore, in the present study, the former was investigated by determining the effects of dopamine’s precursor L-tyrosine on WM in healthy young adults, while isolating cognitive performance related to (1) updating, as well as (2) opening and (3) closing the gate to WM.

To empirically distinguish these processes, the reference-back paradigm has been introduced (Rac-Lubashevsky & Kessler, 2016a). In this adaptation of the widely-used *N*-back paradigm, a sequence of red and blue stimuli is presented. Subjects must indicate whether each stimulus is the same as or different from the stimulus most recently shown in red. Consequently, blue stimuli (referred to as comparison trials) require maintenance of the current reference stimulus (i.e., the most recent stimulus shown in red). In contrast, red stimuli (referred to as reference trials) require updating (i.e., encoding the currently shown stimulus as the reference for subsequent trials). Given that comparison and reference trials are comparable in behavioral response requirements, the performance difference on these trials provides a measure of how efficiently WM content is updated. The behavioral cost associated with transitioning between these trials provides a measure of how efficiently the gate to WM is opened or closed: switching from comparison to reference trials requires opening the gate (relative to consecutive reference trials where the gate can presumably remain open), whereas switching from reference to comparison trials requires closing the gate (relative to consecutive comparison trials where the gate can presumably remain closed).

Converging evidence supports the validity of the reference-back paradigm as a tool to measure and isolate WM processes. First, individual differences in reference-back performance positively correlated with performance on the *N*-back paradigm (Rac-Lubashevsky & Kessler, 2016a), consistent with the idea that both tap into WM. Second, trials requiring WM updating and gate switching elicited an increase in spontaneous eye blink rate (Rac-Lubashevsky, Slagter, & Kessler, 2017)—a presumed marker of dopamine activity in BG (Jongkees & Colzato, 2016), which implements the gating mechanism (Hazy et al., 2006). Third, an fMRI study showed BG activation only when switching from comparison to reference trials (Nir-Cohen, Kessler, & Egner, 2019), suggesting this particular condition indeed involves the gating mechanism in BG (Chatham & Badre, 2015; Frank et al., 2001; Hazy et al., 2006; O’Reilly, 2006). Lastly, an EEG study revealed that transitions from reference to comparison trials elicited increased midfrontal theta power (Rac-Lubashevsky & Kessler, 2018), consistent with a need for distractor-resistant maintenance of WM. In summary, the reference-back paradigm is well-suited to disentangle component processes of WM.

Based on this existing literature and neurocomputational models suggesting a dopamine-driven gating mechanism in BG (Chatham & Badre, 2015; Frank et al., 2001; Hazy et al., 2006; O’Reilly, 2006), L-tyrosine was expected to promote reference-back performance related to gate opening by enhancing phasic dopamine activity in BG. Given that L-tyrosine’s effect on the dopaminergic system might not be selective with respect to the neural networks it affects, and based on literature showing that higher dopamine levels in PFC stabilize WM representations (Durstewitz & Seamans, 2008), it was also considered possible for L-tyrosine to promote WM maintenance and gate closing, by enhancing neural gain in PFC. This improvement in maintenance could possibly also result in less efficient WM updating. Alternatively, it is possible that a non-selective facilitation of gate opening due to L-tyrosine could interfere with the maintenance of task-relevant information, by increasing the chance that irrelevant input is gated into WM. To investigate these potential effects of L-tyrosine, subjects also completed a Stroop task. Recent neurocomputational modelling indicates that higher neural gain in PFC reduces response interference in Stroop-like paradigms (Musslick, Jang, Shvartsman, Shenhav, & Cohen, 2018). Therefore, it was expected that if L-tyrosine increases neural gain then this would be reflected in reduced congruency effects in a Stroop task. Alternatively, if L-tyrosine were to non-selectively promote input-gating then this would facilitate intrusion of WM by the task-irrelevant word meaning, thereby producing larger congruency effects.

Lastly, because the relationship between dopamine activity and WM is not linear but follows an inverted-U curve (Cools & D’Esposito, 2011), two potential predictors of individual differences in the effects of L-tyrosine were investigated: first, baseline WM span was considered, as it has been positively correlated with dopamine synthesis capacity in BG (Cools, Gibbs, Miyakawa, Jagust, & D’Esposito, 2008) and predicted the effect of dopaminergic manipulations on WM (Frank & O’Reilly, 2006; Gibbs & D’Esposito, 2005b). Second, baseline task performance (in a placebo condition) was considered, as it frequently predicts the behavioral effects of catecholaminergic manipulations (Cools & Robbins, 2004; Jepma et al., 2016, 2018) and therefore may indicate an individual’s baseline placement on the inverted-U curve relating dopamine activity and WM performance.

## Methods

### Subjects

Forty-five young adults (25 females) were recruited to participate in a study on L-tyrosine and cognition. One subject was excluded from all analyses due to them using marijuana in the days before the study; two subjects had technical problems while performing the reference-back task; four subjects had technical problems during the Stroop task; three subjects did not fill in an affect grid to measure self-reported mood at all time points. This left 42, 40, and 41 subjects for analysis of the reference-back task, the Stroop task, and the affect grid, respectively.

Subjects met the following criteria: (1) aged 18 to 30 years; (2) no use of drugs or psychoactive medication, nor smoking more than one cigarette per day; (3) no history of psychiatric or neurological conditions; (4) no colorblindness; and (5) women had to be using hormonal contraception, to limit confounding fluctuations in hormone and dopamine levels related to the menstrual cycle (Czoty et al., 2009; Jacobs & D’Esposito, 2011). Subjects were instructed to refrain from caffeine intake in the 3 hours before participation.

### Study design

The study consisted of two sessions of 2 h each, which were separated by 1 week. In both sessions, subjects were administered either 2 g of L-tyrosine or an inert placebo (microcrystalline cellulose) in powder form that was dissolved in 400 mL of orange juice (Colzato, Jongkees, Sellaro, & Hommel, 2013; Jongkees et al., 2017). The order of the L-tyrosine and placebo sessions was counterbalanced across subjects. Administration of L-tyrosine and placebo occurred in a double-blind manner, with neither the experiment leader nor the subject knowing when L-tyrosine or placebo was administered.

Extensive literature indicates that L-tyrosine is effective at inducing a modest increase in catecholaminergic activity (Jongkees et al., 2015). For example, administration of L-tyrosine previously increased plasma levels of dopamine’s metabolite homovanillic acid in Parkinson’s patients (Growdon, Melamed, Logue, Hefti, & Wurtman, 1982), as well as plasma levels of noradrenaline in healthy volunteers (Kishore et al., 2013). A broad literature has shown that L-tyrosine can reverse stress-induced impairment in WM performance (Jongkees et al., 2015), as well as enhancing performance without an external stressor when WM load is sufficiently high (Colzato et al., 2013; Jongkees et al., 2017; Thomas, Lockwood, Singh, & Deuster, 1999). L-tyrosine also has been shown to modulate the effect of noninvasive brain stimulation (Jongkees et al., 2017) in a manner that conceptually mirrored the effects of L-dopa (Kuo, Paulus, & Nitsche, 2008), as well as preexisting baseline differences in dopamine activity as predicted by the COMT Val158Met polymorphism (Plewnia et al., 2013).

### Procedure

Upon arrival at the first session, oral and written informed consent was obtained from all subjects. Thereafter, demographic information was collected. Subjects then rated their current mood state on a 9x9 affect grid consisting of two orthogonal dimensions: subjective arousal and pleasure (Russell, Weis, & Mendelsohn, 1989). Scores on each dimension could range from −4 to +4, with 0 indicating neutral mood. Afterwards, subjects were administered either L-tyrosine or placebo. They then had to wait 1 h for plasma L-tyrosine levels to peak (Glaeser, Melamed, Growdon, & Wurtman, 1979). Immediately after the administration, subjects completed the WM span test and a health questionnaire to confirm participation criteria a second time. Exactly 1 h after administration, subjects again filled in the affect grid and then performed the reference-back and Stroop tasks. The order of the two tasks was constant for each individual subject but counterbalanced across subjects and L-tyrosine/placebo session order. The affect grid was completed a final time at the end of the session.

For exploratory purposes, during the 1-h waiting period subjects additionally completed some questionnaires: the behavioral inhibition/avoidance scales (BIS/BAS); the sensitivity to punishment and sensitivity to reward questionnaire (SPSRQ); and the 21-item depression, anxiety, and stress scale (DASS-21). They also completed another affect grid, after which half of them performed a Raven’s Progressive Matrices (RPM) for 10 min while the other half completed the Advanced Progressive Matrices (APM). After completing the reference-back and Stroop tasks, subjects completed the following two additional tasks in a fixed order: a temporal discounting (TD) task, and the IOWA gambling task. Only the reference-back and Stroop task results will be focused on, which were the main measures of interest, whereas the results of these exploratory measures are not reported here or elsewhere. Importantly, the baseline-dependent effects of L-tyrosine reported later on are not confounded by the order in which subjects completed the reference-back and Stroop tasks, nor whether they performed the RPM or APM during the waiting period (see Supplementary Information for further analyses).

The second session was similar to the first, except that subjects who previously received L-tyrosine now received placebo, and vice-versa. The following measures were omitted in the second session: demographics, WM span, all questionnaires, and the Iowa Gambling task.

### Working memory span

Subjects completed an auditory forward and backward span test. Number sequences were presented using audio clips, with sequence length ranging between 3 to 8 numbers in the forward condition and 2 to 7 numbers in the backward condition. After each clip, subjects had to repeat the sequence in the same (forward condition) or reversed (backward condition) order as presented. Subjects were instructed to maintain the same pace as the audio clip, which was approximately one number per second. Every other trial the sequence length increased by one number. Both conditions were terminated when subjects made a mistake on two consecutive trials or after two trials of the maximum sequence length in that condition. The forward condition was always completed first. WM span scores were calculated as the sum of correct trials across the forward and backward condition (range 0-24).

The mean WM span score was 14.14 (SD = 2.75). The median was 14, based on which the sample was split in a low-capacity group scoring either below (*N* = 19) or at the median (*N* = 6) vs a high-capacity group scoring above the median (*N* = 19). As expected because oral L-tyrosine administration takes some time to elevate plasma L-tyrosine levels (Glaeser et al., 1979), there was no significant difference in span score between subjects who performed the task immediately after placebo versus L-tyrosine in their first session (*M* = 13.45 vs. 14.71, respectively), *t*(41.65) = −1.549, *p* = 0.129.

### Reference-back paradigm

**Fig. 1:**
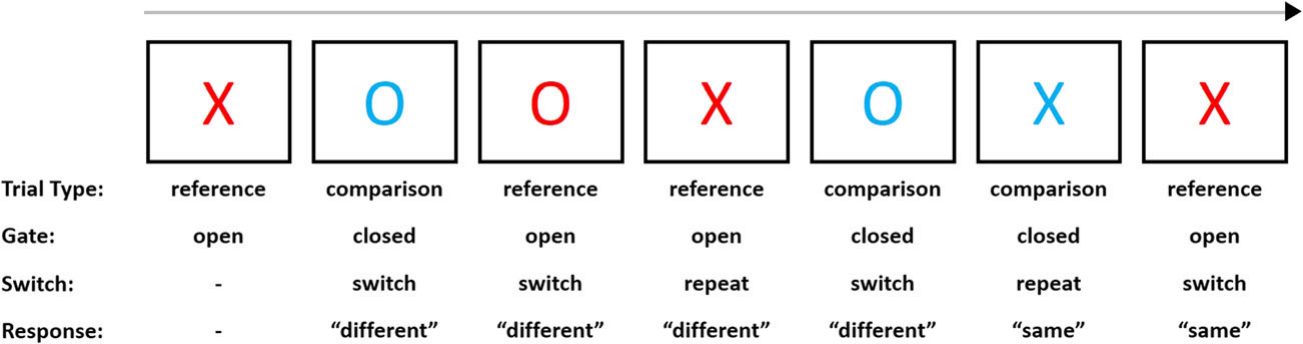
Schematic depiction of an example sequence in the reference-back task. Subjects are instructed to indicate for each letter whether it is the same as or different from the letter most recently shown in red. Figure adapted from Rac-Lubashevsky and Kessler (2016a)

To empirically distinguish component processes of WM, subjects completed the recently introduced reference-back paradigm (see Fig. 1 for a schematic depiction; see also Rac-Lubashevsky & Kessler, 2016a). Each trial started with a white fixation cross in the center of a black screen. After 1 s, the fixation cross was replaced with the letter X or letter O in the color red (on reference trials) or blue (on comparison trials). Subjects had to report whether each letter was the same as or different from the most recent letter shown in red using the “q” and “p” buttons on a QWERTY keyboard, respectively. Stimuli were response-terminated. Subjects were instructed to respond as fast and accurately as possible.

Each block started with a red X or O presented on the screen for 2 s, which served as the reference stimulus for the first trial. For all trials within each block, the stimulus color (red vs. blue) and identity (X vs. O) was completely randomized so that each trial had a 50% chance of being a reference or comparison trial, a switch or repeat trial (i.e., an alternation vs. repetition of trial type from the previous trial), and requiring the “same” or “different” response. Subjects completed 2 practice blocks followed by 12 experimental blocks of 40 trials each. The task took approximately 20 min to complete.

The three dependent measures of interest included the behavioral costs associated with (1) updating of WM content, and (2) opening, as well as (3) closing the gate to WM. Both reference and comparison trials require a stimulus-matching decision, but reference trials additionally require WM updating. Updating cost was calculated as the performance difference on reference minus comparison trials, specifically repeat trials to limit the influence of switching the WM gate. Gate opening cost was calculated as the performance difference on reference switch minus reference repeat trials. Both require an open gate to update WM content, but switch trials additionally require opening the gate when it needed to be closed on the previous trial. Conversely, gate closing cost was calculated as the performance difference on comparison switch minus comparison repeat trials. Both require a closed gate for WM maintenance, but switch trials additionally require closing the gate when it needed to be open on the previous trial.

### Stroop paradigm

To assess the potential effect of L-tyrosine on neural gain in PFC and potentially unselective input-gating, subjects completed a Stroop task. Each trial started with a white fixation cross presented in the center of a black screen. After 500 ms, the fixation cross was replaced with the word yellow, blue, green, or red in one of these four colors. The stimuli were presented until a response was given; subjects had to indicate the print color of the word while ignoring the word meaning, by respectively pressing the “z” or “x” buttons with their left hand and the “>” or “?” buttons with their right hand on a QWERTY keyboard. The next trial started immediately after a response was given. Half of all trials were congruent (word meaning and print color matched) and half were incongruent (word meaning and print color did not match). Subjects were instructed to respond as fast and accurately as possible.

Subjects first performed a practice block of 20 trials wherein they had to indicate the print color of a meaningless string (XXXXX). Thereafter, they completed 4 experimental blocks of 80 trials each. The task took approximately 10 min to complete. Congruency effects were calculated as the performance difference on incongruent minus congruent trials.

### Statistical analyses

To check whether L-tyrosine affected subjective mood states that could influence task performance, the arousal and pleasure scores from the affect grid were submitted to separate repeated measures ANOVA (rmANOVA) with Time (baseline vs. 1 h after L-tyrosine/placebo vs. end of the session) and Treatment (L-tyrosine vs. placebo) as within-subject factors.

For the reference-back data, the first trial of each block was excluded. Mean error rate (ER) and median reaction time (RT) was then calculated for each of the four conditions of the task (reference switch and repeat, comparison switch and repeat). For RT data, both error and post-error trials were excluded. Outlier subjects were identified separately for the four conditions of the task in the L-tyrosine and placebo sessions, based on three times the interquartile range. To investigate potential main effects of L-tyrosine on performance, ER and RT were submitted to separate rmANOVAs with Trial Type (reference vs. comparison), Switch (switch vs. repeat), and Treatment (L-tyrosine vs. placebo) as within-subject factors. For a more direct test of L-tyrosine’s effect on specific component processes of WM, planned paired sample *t* tests were performed comparing the L-tyrosine and placebo conditions on updating cost, gate opening, and gate closing (only in RT as ER was very low at approximately 0.05).

Similarly, for the Stroop data, the first trial of each block was excluded. Mean ER and median RT was then calculated for incongruent and congruent trials. For RT data, both error and post-error trials were excluded. Outlier subjects were identified separately for congruent and incongruent trials preceded by congruent and incongruent trials in the L-tyrosine and placebo sessions, based on three times the interquartile range. To investigate potential main effects of L-tyrosine on performance, ER and RT were submitted to separate rmANOVAs with Congruency (congruent vs. incongruent) and Treatment (L-tyrosine vs. placebo) as within-subject factors.

To examine WM span as moderator of L-tyrosine’s effects, the abovementioned rmANOVAs were repeated including the median split grouping variable (below or at vs. above the median WM span) as between-subjects factor.

To investigate whether L-tyrosine had baseline-dependent effects, it was examined whether subjects’ behavioral response to L-tyrosine depended on their performance in the placebo session. To do this, first across-subject Pearson r correlations were calculated between placebo performance and L-tyrosine minus placebo performance for the following outcome measures: updating cost, gate opening, and gate closing in the reference-back task, as well as congruency effects in the Stroop task. It is important to consider that such correlations will typically be significant and negative not only because of potential baseline-dependent effects of a manipulation, but also as a result of regression to the mean (Kelly & Price, 2005). Additionally, the correlation of these measures is likely to be negative because the variance of the placebo scores is present in both measures (i.e., the placebo scores and the L-tyrosine minus placebo difference scores) but with opposite signs. To address this shortcoming of the correlational analysis and to determine whether L-tyrosine’s baseline-dependent effects were stronger than expected based on regression to the mean, a test of equality of between-subject variance in the L-tyrosine and placebo conditions was conducted. Critically, if low-performing subjects improved more than expected based on regression to the mean and/or high-performing subjects decline more (as predicted by an inverted-U curve relation between dopamine activity and WM performance), then this convergence to the mean would result in reduced variability in performance between subjects in the L-tyrosine compared with placebo condition (Kelly & Price, 2005). Importantly, because the order of the placebo and L-tyrosine conditions was counterbalanced across subjects, a reduced variance within the L-tyrosine condition cannot be attributed to factors such as a practice or order effect, nor regression to the mean. A potentially reduced variance in the L-tyrosine versus placebo condition was tested using a one-tailed Pitman’s test of equality of variance in paired samples (Pitman, 1939), as implemented in the R package PairedData.

## Results

### L-tyrosine does not affect self-reported mood

For subjective arousal, there were no main effects of Time or Treatment, nor a two-way interaction, *p*s ≥ 0.213. This suggested stable levels of perceived arousal throughout the experiment that did not differ between placebo and L-tyrosine (*M*_Baseline_ = −0.61, *M*_Before tasks_ = −0.20, *M*_After tasks_ = −0.40). Similarly, for subjective pleasure, there were no main effects of Time or Treatment, nor a two-way interaction, *p*s ≥ 0.874. This suggested stable levels of perceived pleasure throughout the experiment that did not differ between placebo and L-tyrosine (*M*_Baseline_ = 1.39, *M*_Before tasks_ = 1.34, *M*_After tasks_ = 1.29). Consistent with previous literature (Jongkees et al., 2015), L-tyrosine did not impact self-reported mood.

### Replication of basic effects in the reference-back task

Before analyzing the effect of L-tyrosine on reference-back performance, an analysis of performance only in the placebo session was conducted to determine whether the present study replicated key performance patterns in the reference-back task as reported in previous studies (Rac-Lubashevsky & Kessler, 2016a, b). Descriptive statistics of all task conditions are presented in Table 1. Three outlier subjects were identified based on three times the interquartile range of task performance. While these subjects were excluded from further analyses, it should be noted their inclusion does not qualitatively influence the results involving the L-tyrosine manipulation reported later on.

**Table 1:** Descriptive statistics of performance on the reference-back task

		Error rate				Reaction time			
		L-tyrosine		Placebo		L-Tyrosine		Placebo	
Trial type	Gate switch	Mean	SD	Mean	SD	Mean	SD	Mean	SD
Reference	Switch	0.037	0.031	0.040	0.031	716	214	693	215
	Repeat	0.049	0.039	0.054	0.040	652	160	627	129
Comparison	Switch	0.040	0.031	0.043	0.034	639	156	620	146
	Repeat	0.039	0.035	0.038	0.032	556	121	536	112
Updating cost		−0.010	0.039	−0.016	0.032	96	71	91	65
Gate opening		0.012	0.032	0.014	0.036	64	78	65	109
Gate closing		−0.001	0.033	−0.005	0.030	83	58	84	65

The RT data revealed substantially slower responses on reference repeat (*M* = 627 ms) than comparison repeat trials (*M* = 536 ms), reflecting the behavioral cost associated with being in an updating mode (*M* = 91 ms, *t*(38) = 8.79, *p* < 0.001). Responses were also slower on reference switch (*M* = 693 ms) than reference repeat trials *(M* = 627 ms), indicating the behavioral cost of opening the gate to WM (*M* = 65 ms, *t*(38) = 3.75, *p* < 0.001). Similarly, responses were slower on comparison switch (*M* = 620 ms) than comparison repeat trials (*M* = 536 ms), reflecting the behavioral cost of closing the gate to WM (*M* = 84 ms, *t*(38) = 8.10, *p* < 0.001). In sum, the results fully replicated previous studies by revealing large and highly significant behavioral costs of WM updating, gate opening, and gate closing.

### Baseline-dependent effect of L-tyrosine on gate opening, but not updating or gate closing

For ER, there were significant main effects of Trial Type, *F*(1,38) = 4.286, *p* = 0.045, partial-*η*^2^ = 0.101, and Switch, *F*(1,38) = 4.118, *p* = 0.049, partial-*η*^2^ = 0.098. ER tended to be slightly higher on reference than comparison trials (*M* = 0.045 vs. 0.040) and higher on repeat than switch trials (0.045 vs. 0.040). Importantly, there was no main effect of Treatment, *F*(1,38) = 0.641, *p* = 0.428, nor any interaction involving Treatment, all *p*s ≥ 0.523. Adding WM span to the analysis did not produce interactions involving WM Span and Treatment, Trial Type and/or Switch, *p*s ≥ 0.245. The low ER (<0.05) suggests that the ER data are unsuited for measuring WM-related performance, possibly because the task did not enforce a response deadline. For these reasons, further analyses were restricted to RT data.

**Fig. 2:**
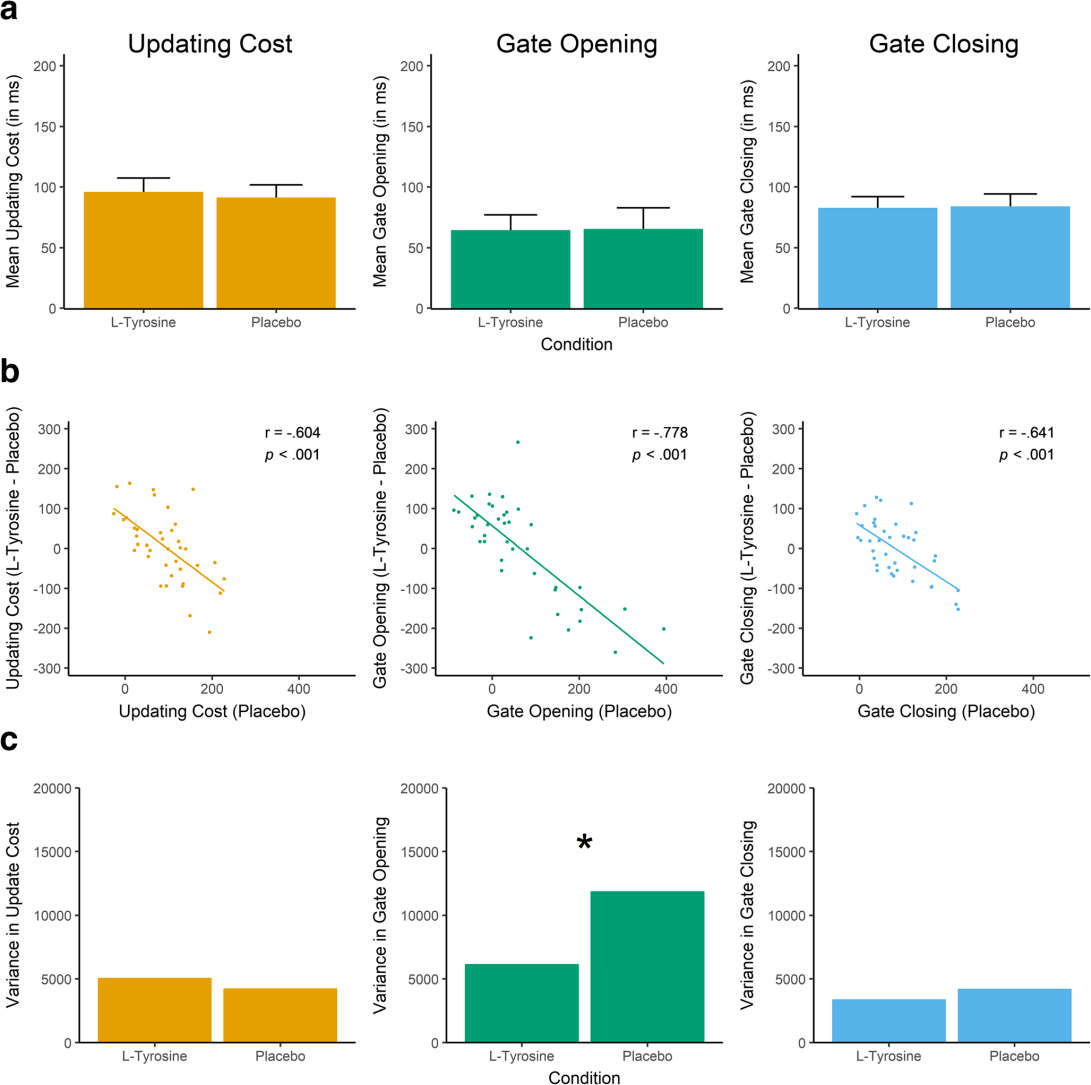
Results from the reference-back task. (**a**) Bar graphs indicating the mean behavioral cost (from RT data) in the L-tyrosine and placebo conditions, including standard error of the mean. (**b**) Pearson r correlations of placebo performance and the L-tyrosine minus placebo performance. On the x-axis, higher scores indicate greater behavioral costs. On the y-axis, positive scores indicate increased cost on L-tyrosine whereas negative scores indicate reduced cost. (**c**) Between-subject variance of the behavioral cost in the L-tyrosine and placebo conditions. *p < 0.05

For RT, there were significant main effects of Trial Type, *F*(1,38) = 125.183, *p* < 0.001, partial-*η*^2^ = 0.767, and Switch, *F*(1,38) = 82.530, *p* < 0.001, partial-*η*^2^ = 0.685, but no Trial Type * Switch interaction, *F*(1,38) = 2.606, *p* = 0.115. When collapsed across the L-tyrosine and placebo condition, RT was higher on reference than comparison trials (*M* = 672 vs. 588) and higher on switch than repeat trials (*M* = 667 vs. 593), with the nonsignificant interaction indicating symmetrical switch costs (switch minus repeat) on reference and comparison trials (*M* = 64 vs. 83). Again, there was no significant main effect of Treatment, *F*(1,38) = 0.545, *p* = 0.465, nor any interaction involving Treatment, all *p*s ≥ 0.740. Adding WM span to the analysis did not produce interactions involving WM Span and Treatment, Trial Type and/or Switch, *p*s ≥ 0.089. As a more direct test of our hypotheses, planned paired *t* tests were conducted to compare L-tyrosine and placebo on updating cost, gate opening, and gate closing scores. As illustrated in Fig. 2A, the analyses revealed no whole-sample effects of L-tyrosine, *p*s ≥ 0.747.

To check for baseline-dependent effects of L-tyrosine, Pearson r correlations were calculated between individual performance in the placebo condition and the difference in performance between the L-tyrosine and placebo conditions. As shown in Fig. 2B, for all three outcome measures, this produced significant negative correlations, which suggested that L-tyrosine reduced costs (i.e., improved performance) in subjects who had high costs on placebo, whereas L-tyrosine increased costs (i.e., impaired performance) in those who had low costs on placebo. Because regression to the mean and mathematical dependency between these performance measures also predict negative correlations, the variability in performance between subjects in the two conditions was compared. Critically, a baseline-dependent effect of L-tyrosine that is stronger than expected based on regression to the mean also should produce less variability in performance between subjects in the L-tyrosine than placebo condition. As illustrated in Fig. 2C, one-tailed Pitman’s test revealed reduced variance in the L-tyrosine condition for gate opening, *t*(37) = 2.072, *p* = 0.023 (*S*^*2*^ = 6,143 vs. 11,874), but not updating cost *t*(37) = −0.552, *p* = 0.708 (*S*^*2*^ = 5044 vs. 4219) or gate closing, *t*(37) = 0.711, *p* = 0.241 (*S*^*2*^ = 3,356 vs. 4,183).

To clarify whether the effect of L-tyrosine on between-subject variability in gate opening performance was driven by a particular modulation of performance on reference switch or reference repeat trials, these trial types were analyzed separately. Both reference switch and repeat trials demonstrated negative Pearson’s r correlations between placebo and L-tyrosine minus placebo performance, r(37) = −0.637, *p* < 0.001 and r(37) = −0.529, *p* < 0.001, respectively. However, Pitman’s test revealed no differences in between-subject variance in the placebo and L-tyrosine condition on reference switch trials, *t*(37) = 0.027, *p* = 0.489, nor on repeat trials, *t*(37) = −1.332, *p* = 0.905. The finding that L-tyrosine affected variance in the performance *difference* on reference switch and repeat trials, whereas performance on either trial separately was simply explained by regression to the mean, indicates a selective modulation by L-tyrosine of that which distinguishes reference switch from repeat trials—the requirement of switching the gate from closed to open.

To further probe a nonlinear effect of L-tyrosine on gate opening performance, the sample was split based on the median gate opening score in the placebo condition. The gate opening scores in the placebo and L-tyrosine conditions were then correlated separately for the below or at median group (*N* = 20) and the above median group (*N* = 19), using Spearman’s rank-ordered correlations to account for small sample sizes. Whereas there was a positive correlation in the below/at median group, r_s_(18) = 0.476, *p* = 0.034, there was a numerically negative but non-significant correlation in the above median group, r_s_(17) = −0.097, *p* = 0.692, with Fisher’s Z test indicating that the former correlation was significantly greater than the latter, Z = 1.766, *p* = 0.039. It should be noted that the two groups were small, a median split might not capture the exact turning point of an inverted-U curve, and these correlations might be biased toward being positive due to individuals differences in performance (e.g., due to different levels of motivation to perform the task) that are shared between the two sessions. Nevertheless, the fact that placebo and L-tyrosine scores were correlated differently depending on the placebo performance provides converging evidence for a nonlinear effect of L-tyrosine on gate opening efficiency.

In summary, the placebo and L-tyrosine conditions did not differ in mean gate opening performance yet the latter had significantly reduced variability in gate opening performance between subjects. Consistent with an inverted-U curve relating dopamine activity and WM performance, these results indicate that L-tyrosine modulated the efficiency of gate opening in a baseline-dependent manner that is stronger than expected based solely on regression to the mean. In contrast, L-tyrosine did not modulate the efficiency with which WM content is updated or the gate to WM is closed.

### No baseline-dependent effect of L-tyrosine on response interference

In Stroop data, there were no outlier subjects based on 3 times the interquartile range of performance. For ER, there was a significant main effect of Trial Type, *F*(1,39) = 64.557, *p* < 0.001, partial-*η*^2^ = 0.623, indicating higher ER on incongruent than congruent trials (*M* = 0.053 vs. 0.030). There was no significant main effect of Treatment, *F*(1,39) = 0.537, *p* = 0.468, nor a Trial Type * Treatment interaction, *F*(1,39) = 0.515, *p* = 0.477. Adding WM span to the analysis did not produce a significant Trial Type * Treatment * WM Span interaction, *F*(1,38) = 0.068, *p* = 0.796.

**Fig. 3:**
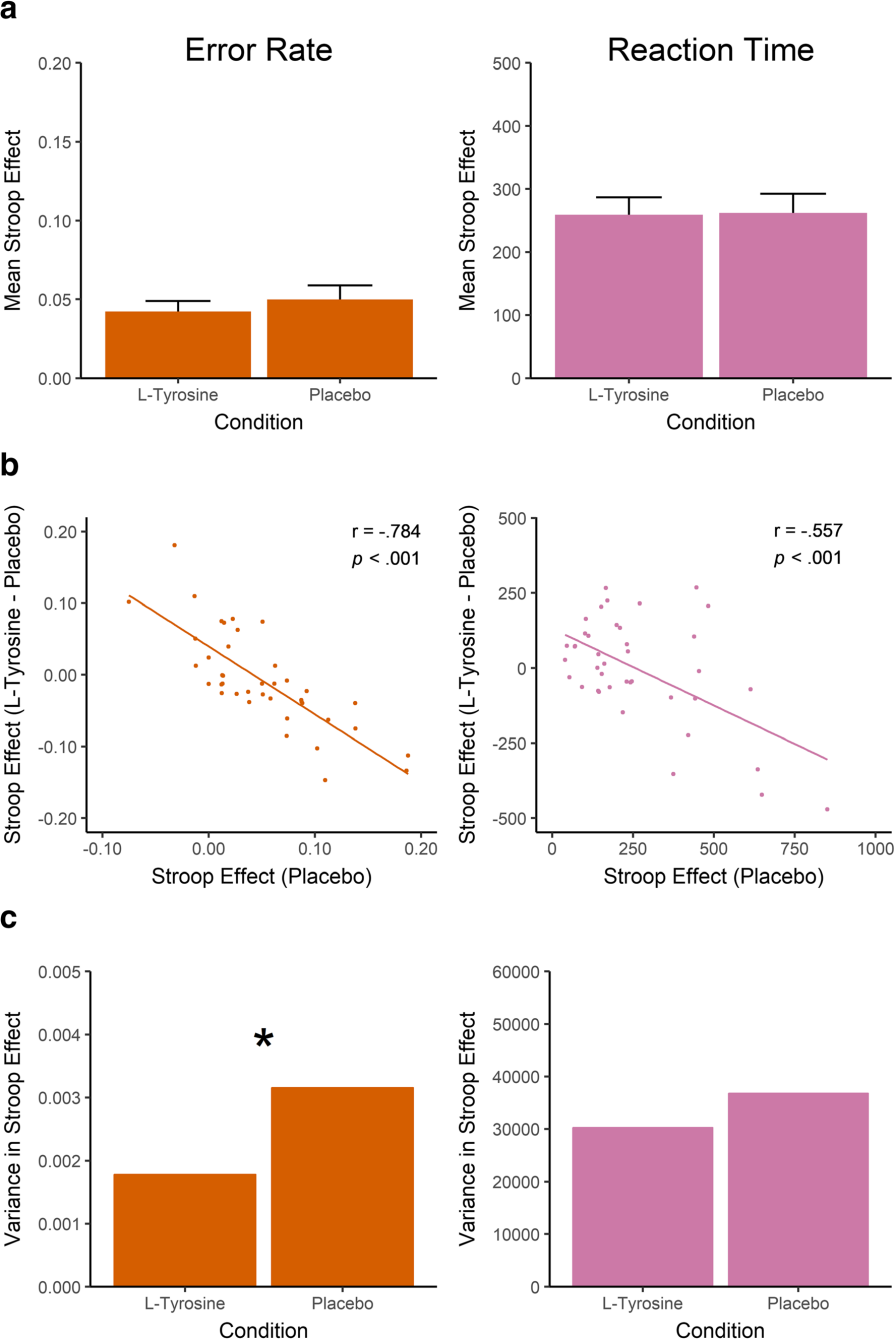
Results from the Stroop task. (**a**) Bar graphs indicating the mean congruency effect in the L-tyrosine and placebo conditions, including standard error of the mean. (**b**) Pearson r correlations of placebo performance and the L-tyrosine minus placebo performance. On the x-axis, higher scores indicate larger congruency effects. On the y-axis, positive scores indicate increased congruency effects on L-tyrosine whereas negative scores indicate reduced congruency effects. (**c**) Between-subject variance of the congruency effect in the L-tyrosine and placebo conditions. *p < 0.05

For RT, there was a significant main effect of Trial Type, *F*(1,39) = 105.524, *p* < 0.001, partial-*η*^2^ = 0.730, indicating higher RT on incongruent than congruent trials (*M* = 788 vs. 657). There was again no significant main effect of Treatment, *F*(1,39) = 0.569, *p* = 0.455, nor a Trial Type * Treatment interaction, *F*(1,39) = 0.010, *p* = 0.921. Adding WM span to the analysis did not produce a significant Trial Type * Treatment * WM Span interaction, *F*(1,38) = 2.094, *p* = 0.156. Figure 3A illustrates the lack of an effect of L-tyrosine on the whole sample level.

To check for baseline-dependent effects of L-tyrosine, the same correlational analyses as reported above were repeated but this time on the congruency scores. As shown in Fig. 3B, both in terms of ER and RT, this produced significant negative correlations. To determine whether the baseline-dependent effect of L-tyrosine is stronger than expected based on regression to the mean and the mathematical dependency between these correlated measures, the variance in performance between the two conditions was again compared. As illustrated in Fig. 3C, one-tailed Pitman’s test of equality of variance in paired samples revealed smaller variance in the L-tyrosine than placebo condition for ER, *t*(38) = 1.787, *p* = 0.041 (*S*^*2*^ = 0.002 vs. 0.003), but not RT *t*(38) = 0.718, *p* = 0.239 (*S*^*2*^ = 36,830 vs. 30,272). However, considering that ER in the Stroop task was extremely low (≈0.05) and thus may constitute an unreliable measure of L-tyrosine’s effects on task performance, there appears to be only tentative evidence for a baseline-dependent effect of L-tyrosine on congruency effects in terms of ER, whereas it had no effect on RT.

## Discussion

The present study investigated whether WM updating and gating were differentially affected by the administration of dopamine’s precursor L-tyrosine. In brief, none of the analyses revealed whole-sample effects of L-tyrosine on updating, gate opening, or gate closing, with or without WM span as moderating factor. However, the results indicated a reliable baseline-dependent effect of L-tyrosine on the efficiency with which the gate to WM is opened, but not updating or gate closing. Specifically, the placebo and L-tyrosine conditions did not differ in mean gate opening performance, yet the latter condition demonstrated markedly reduced variability in performance between subjects. This pattern of results indicates that L-tyrosine facilitated gate opening in individuals who performed poorly on placebo, whereas it was hindered in those who already performed well, thereby contracting the distribution of performance in the L-tyrosine condition while the mean performance remained unchanged as compared to placebo. Importantly, the reduction in variability between subjects due to L-tyrosine cannot be accounted for by regression to the mean (Kelly & Price, 2005) nor an order/practice effect because the order of the placebo and L-tyrosine conditions was counterbalanced across subjects. The interpretation of L-tyrosine modulating the ability to open the gate to WM in a baseline-dependent manner is discussed further below.

The baseline-dependent effect of L-tyrosine on gate opening is remarkably consistent with the well-established inverted-U curve relating dopamine activity and WM (Cools & D’Esposito, 2011), as well as previous literature showing state-dependent effects of L-tyrosine on cognitive control (Jongkees et al., 2015). Converging evidence that effects of L-tyrosine depend on baseline dopaminergic state in particular comes from a recent (preprinted) study in healthy older adults showing that L-tyrosine’s effect on the willingness to exert cognitive control was predicted by individual trait impulsivity (Froböse, Westbrook, Bloemendaal, Aarts, & Cools, 2019), a correlate of dopamine activity in BG (Buckholtz et al., 2011). Together, these findings indicate that L-tyrosine can modulate both the ability and motivation to engage in cognitive control in a state-dependent manner.

Such nonlinear results could be at least partially accounted for by a shift in an individual’s ratio between phasic (stimulus-driven) and tonic (spontaneous) dopamine release in BG (Grace, 1991, 2016). One hypothesis is that in subjects with already optimal levels of phasic dopamine activity (and presumably efficient gating), augmenting dopamine release with L-tyrosine does not result in further occupation of postsynaptic dopamine receptors. Instead, there might be a net increase in occupancy of presynaptic, inhibitory autoreceptors that reduce the intensity of the phasic response (Grace, 1991, 2016). According to prominent neurocomputational models (Chatham & Badre, 2015; Frank et al., 2001; Hazy et al., 2006; O’Reilly, 2006), a blunted phasic response should hinder gate opening. In contrast, in subjects with suboptimal phasic dopamine activity (and presumably inefficient gating), L-tyrosine might shift the phasic/tonic ratio in favor of phasic activity, leading to a net increase in postsynaptic dopamine activity and thereby facilitating gate opening (see Jepma et al., 2016, for a similar account of a catecholaminergic manipulation in terms of shifting the phasic/tonic ratio of activity).

Another, not mutually exclusive hypothesis relates to the finding in animal research that low dose L-tyrosine (25 mg/kg) effectively increased L-tyrosine hydroxylation in a sustained manner, whereas higher dosage of L-tyrosine (50 mg/kg) triggered end-product inhibition (Tam & Roth, 1997). Perhaps similar differential effects on L-tyrosine hydroxylation can occur with a fixed dosage in individuals with respectively lower versus higher baseline levels of dopamine activity, thereby contributing to L-tyrosine’s baseline-dependent effects.

Regarding the baseline-dependent effect of L-tyrosine on gate opening, one might raise the concern that this finding is driven by unusual performance in the placebo condition rather than a selective modulation of performance in the L-tyrosine condition. To elaborate, less variability between subjects in gate opening performance in the L-tyrosine than placebo condition is the main evidence of L-tyrosine’s baseline-dependent effect beyond regression to the mean. However, inspection of Fig. 2C might suggest that rather than variance in the L-tyrosine condition being reduced, the variance across subjects in the placebo condition was unusually high compared with the other behavioral measures. This is, however, not unusual. An unpublished, single-session study performed by the author (*N* = 43) used an identical version of the reference-back paradigm but without any manipulation, such as L-tyrosine administration. That study also revealed significantly higher variance in gate opening relative to updating and gate closing performance (see Supplementary Information for more detail). Similar behavioral results have been reported recently, which consistent with the present study’s results, indicate that high variability in reference switch trials relative to the other trials contributes to the greater variance in gate opening performance (Nir-Cohen et al., 2019). Rac-Lubashevsky and Kessler (2016a) also reported numerically greater variance in gate opening performance relative to the other contrasts, although their numerical difference might not be significant due to methodological differences, such as a lower trial type switching relative to the present study (25% vs. 50%). Importantly, within the context of the present study, these results highlight that the difference in gate opening variance between the L-tyrosine and placebo condition is not driven by unusual performance under placebo. Instead, the selectively reduced variance supports a baseline-dependent effect of L-tyrosine on gate opening performance beyond what is expected by regression to the mean.

In addition to investigating the effect of L-tyrosine on WM processes, it also was investigated whether L-tyrosine would modulate congruency effects in a Stroop task. The reasoning behind this was that a nonselective facilitation of input-gating to WM could result in increased distractibility by task-irrelevant input (Hazy et al., 2006), which would lead to an increase in Stroop congruency effects. Alternatively, reduced rather than increased distractibility by task-irrelevant input could have been expected due to elevated tonic dopamine levels that would increase neural gain in PFC (Durstewitz & Seamans, 2008). Indeed, animal research showed that the high basal firing rate of mesoprefrontal neurons makes them especially sensitive to the availability of L-tyrosine (Tam, Elsworth, Bradberry, & Roth, 1990), supporting the hypothesis that L-tyrosine could modulate neural gain in PFC. While the results did point to a baseline-dependent of L-tyrosine on congruency effects in terms of errors, evidenced by reduced ER variance in the L-tyrosine condition, it should be emphasized that average ER was very low (≈0.05) and thus might be unsuited for detecting effects of L-tyrosine on performance. Therefore, the Stroop data should be interpreted with caution. One recent study showed that L-tyrosine did modulate response interference in a Simon but not Flanker task, indicating that reliability of L-tyrosine’s effects on response interference might differ based on the specific paradigm and whether it involves stimulus-response or stimulus-stimulus conflict (Stock, Colzato, & Beste, 2018). However, it should be noted that the sample size was smaller than in the present study (*N* = 22-25 vs. 40), the order of the Simon and Flanker tasks was not counterbalanced, and the authors did not investigate potential baseline-dependent effects of L-tyrosine. It is thus highly necessary to further investigate this topic before drawing conclusions on the potential effects of L-tyrosine on response interference, especially as a putative marker for its effects on neural gain.

The present findings have important and broad implications for the extensive literature on WM. It was shown that a dopaminergic manipulation differentially affected WM updating and gating—specifically gate opening but not closing. In doing so, the present study highlights that a dopaminergic manipulation of—or disturbance in—WM should be interpreted with care regarding the mechanism underlying these effects. For example, there has been great interest in using techniques such as transcranial direct current stimulation (tDCS) to modulate WM in both healthy and clinical populations (Plewnia, Schroeder, & Wolkenstein, 2015). Although tDCS has relatively direct effects on cortical excitability (Nitsche et al., 2003; Nitsche & Paulus, 2000), there is evidence it also indirectly modulates dopamine activity in BG (Fonteneau et al., 2018; Meyer et al., 2019; Tanaka et al., 2013). The present study thus raises the question whether tDCS acts on WM via a modulation of more prefrontal-based updating/maintenance, BG-based gating efficiency, or both. The answer would allow for clearer predictions regarding the populations that stand to benefit from tDCS in terms of WM function, which is a critical step toward reducing the strong heterogeneity in findings with tDCS. The present results also are relevant to other fields of research, including those investigating WM deficits in neuropsychiatric disorders and particularly ones with a dopaminergic pathophysiology. For example, major depression is characterized by a strong disturbance in WM (Banich et al., 2009; Foland-Ross & Gotlib, 2012; Gotlib & Joormann, 2010; Joormann, 2018; Kaiser et al., 2015). This is likely related to an initial hyperactivity of the dopaminergic system that eventually leads to long-term downregulation of dopamine activity in BG, thereby contributing to anhedonia and lack of motivation (Grace, 2016). Based on the theoretical framework of the present study, this would presumably also lead to a particular deficit in control over input-gating. This raises the possibility that the impaired clearing of WM from negative content (Zetsche et al., 2018) is actually related to a deficit in gating rather than an issue with the updating/removal process per se. These results directly illustrate the importance of carefully distinguishing between component processes of WM within many branches of the WM literature.

The interpretation that L-tyrosine affected gate opening via a modulation of dopamine activity in BG is notably consistent with a recent fMRI study showing that gate opening was the only behavioral contrast in the reference-back task to selectively activate the BG (Nir-Cohen et al., 2019). However, a question that remains unanswered is to what extent the state of the WM gate is indeed persistent over time; further research is necessary to clarify in detail how this supposed persistence contributes to the gate opening and closing measures in the reference-back paradigm. For simplicity’s sake, this and previous studies have mostly reasoned as if the WM gate maintains its state across trials when the environment does not require it to change state Rac-Lubashevsky & Kessler, 2016a, b). This would avoid the cost of unnecessarily switching the gate, which is taken to account for the better performance on consecutive reference or comparison trials where the gate could stay open or closed, respectively. Rather than the gate being either fully open or closed, it has been speculated previously (Kessler, 2017) that it also is possible that the likelihood and/or extent of gate opening/closing increases across consecutive reference/comparison trials, in order to balance the cost of switching the gate with the need to update/shield WM content. To effectively investigate this hypothesis, it is necessary to utilize study designs better suited to examine performance on more than one consecutive reference or comparison trial and supplement findings with neurocomputational modelling.

It also is necessary to clarify in more detail the exact neural processes involved in the gate opening and closing measures. Candidate processes underlying these measures include the efficiency/extent of thalamic disinhibition, the efficiency with which the PFC is toggled between its high and low gain states, or perhaps more simply the level of prefrontal gain in general (Hazy et al., 2006). A role for thalamic disinhibition would be consistent with the observation that gate opening in the reference-back was related to activation of the thamalus (Nir-Cohen et al., 2019). A role for prefrontal gain would fit with recent neurocomputational modelling of task-switching performance, suggesting that subjects adjust their overall gain levels according to the situational demand for more task switches or repetitions (Musslick, Bizyaeva, Agaron, Leonard, & Cohen, 2019); in a similar fashion, subjects might adjust prefrontal gain according to the overall and perhaps trial-by-trial changes in demand for WM updating versus maintenance.

As is often the case in studies manipulating the dopaminergic system, it should be acknowledged that such manipulations are likely to affect the noradrenergic system as well. There is indeed evidence that L-tyrosine can modulate noradrenaline activity, as it has affected the P300 event-related potential (Kishore et al., 2013), a putative marker of phasic noradrenaline activity in the locus coeruleus (Nieuwenhuis, Aston-Jones, & Cohen, 2005). Although the present findings are remarkably consistent with studies that related gate switching in the reference-back task to a dopaminergic marker (Rac-Lubashevsky, Slagter, & Kessler, 2017) and activity in BG (Nir-Cohen et al., 2019), an explanation of the present results in terms of noradrenaline cannot be definitively ruled out. Future research therefore should include and control for measures of noradrenaline activity, for example pupil dilation (Joshi, Li, Kalwani, & Gold, 2016; Murphy, O’Connell, O’Sullivan, Robertson, & Balsters, 2014; Reimer et al., 2016) or salivary alpha amylase levels (Warren et al., 2019; Warren, van den Brink, Nieuwenhuis, & Bosch, 2017).

On a more methodological note, the results underscore the importance of considering regression to the mean when analyzing baseline-dependent effects in repeated measurements. Baseline performance and the change in performance on any repeated measurement are likely to be negatively correlated based purely on regression to the mean (Kelly & Price, 2005), yet such patterns can easily be mistaken as evidence for inverted-U shaped effects. Literature on catecholaminergic manipulations often still do not consider this confounding factor. It is therefore important for future research to acknowledge and control for regression to the mean in order to avoid overestimating the efficacy of a manipulation or intervention. Furthermore, it is important to note that when correlating performance in condition A (e.g., placebo) with the performance difference between session B minus A (e.g., L-tyrosine minus placebo), then the variance of A is included in both measures but with opposite sign. This mathematical dependency between the data guarantees a negative correlation between the two measures, rendering it necessary to include additional analyses to determine whether the baseline-dependency is stronger than expected based on this mathematical relationship between the two measures. One simple method for achieving this while avoiding the issue of regression to the mean is to test for equality of variances between the repeated measurements (Kelly & Price, 2005), which was done in the present study. If a low-performing subject improves more than expected based only on regression to the mean, and/or a high-performing subject is impaired more than expected, then variance across subjects in the experimental condition should be reduced compared with the baseline condition. In the present study, this was the case only for gate opening performance in the L-tyrosine relative to placebo condition, pointing toward a robust baseline-dependent modulation of one’s ability to open the gate to WM.

Lastly, it should be noted that future research would benefit from additional manipulation checks to determine whether L-tyrosine successfully elevated catecholamine activity and whether this response might vary between individuals, thereby contributing to individual differences in the cognitive-behavioral response to L-tyrosine administration. Two possible markers that have been used for such purposes previously, both derivable from blood samples, are plasma catecholamine levels, which were shown to be elevated by L-tyrosine administration (Kishore et al., 2013), and prolactin level, which has been used to asses drug effects on dopamine in BG and WM performance (Frank & O’Reilly, 2006).

## Conclusions

This study has provided evidence that dopamine’s precursor L-tyrosine modulates the efficiency of opening the gate to WM, but not updating its content or closing the gate. This supports a dopaminergic basis specifically for the process of gate opening, as has been previously proposed by neurocomputational modelling work. The baseline-dependent manner in which L-tyrosine affected gate opening highlights the need to consider individual differences in studies on L-tyrosine/dopamine and WM. Furthermore, the results underscore the importance of empirically distinguishing between WM updating and gating—distinct component processes of adaptive WM that are now shown to be differentially sensitive to a dopaminergic manipulation.

## Open practices statement

Data and R scripts for preprocessing, analysis, and figures are available for download on dataverse.nl (https://hdl.handle.net/10411/JWILQU). The experiment was not preregistered.

## Electronic supplementary material

ESM 1 (DOCX 22.4 kb)
