# Supplementary material for: Baseline-dependent effect of dopamine’s precursor L-tyrosine on working memory gating but not updating
Source: Cogn Affect Behav Neurosci. 2020 Mar 4;20(3):521–35. doi: 10.3758/s13415-020-00783-8 (PMC7266860; doi:10.3758/s13415-020-00783-8)
Supplement: Supplementary file 1 — (DOCX 22.4 kb) [file 13415_2020_783_MOESM1_ESM.docx]

**Supplementary Material**

*Effect of L-tyrosine on gate opening is not confounded by treatment or task order nor Raven version*

It was investigated whether the baseline-dependent effect of L-tyrosine on performance in the reference-back task was perhaps confounded by individual differences in the order of treatment (L-tyrosine vs placebo), the order of the two main tasks (reference-back and Stroop task), or the version of the Raven performed in the 1 h waiting period (RPM vs APM). To anticipate the conclusion of these analyses, none indicate that L-tyrosine’s impact on gate opening is explained by these factors.

First, a rmANOVA was conducted on RT in the reference-back with Trial Type (reference vs comparison), Switch (switch vs repeat), and Treatment (L-tyrosine vs placebo) as within-subject factors and Treatment Order (placebo first vs L-tyrosine first) as between-subject factor, see Table S1. This revealed significant interactions between Treatment Order and Treatment, as well as interactions further involving Trial Type or Switch, *p*s < .001. These interactions involving Treatment Order and Treatment reflected a session order (i.e., practice) effect whereby RT was on average higher in the first than second session: for subjects receiving placebo first, mean RT was higher in the placebo than L-tyrosine condition (*M* = 685 vs 552, *p* < .001), whereas for subjects receiving L-tyrosine first, mean RT was higher in the L-tyrosine than condition (*M* = 717 vs 562, *p* < .001).

| *Table S1.* Analysis of treatment order and reference-back performance (in RT) | | | | |
| --- | --- | --- | --- | --- |
| **Effect** | **DF** | ***F*** | ***p*** | ***η*^2^*_p_*** |
| Treatment Order | 1,37 | .292 | .592 | .008 |
| Treatment | 1,37 | .337 | .565 | .009 |
| Trial Type | 1,37 | 120.952 | **< .001** | .766 |
| Switch | 1,37 | 80.834 | **< .001** | .686 |
| Treatment Order * Treatment | 1,37 | 60.983 | **< .001** | .622 |
| Treatment Order * Trial Type | 1,37 | .050 | .824 | .001 |
| Treatment Order * Switch | 1,37 | .174 | .679 | .005 |
| Treatment * Trial Type | 1,37 | < .001 | .999 | < .001 |
| Treatment * Switch | 1,37 | .199 | .657 | .005 |
| Trial Type * Switch | 1,37 | 2.332 | .135 | .059 |
| Treatment Order * Treatment * Trial Type | 1,37 | 36.034 | **< .001** | .493 |
| Treatment Order * Treatment * Switch | 1,37 | 20.503 | **< .001** | .357 |
| Treatment Order * Trial Type * Switch | 1,37 | 1.370 | .249 | .036 |
| Treatment * Trial Type * Switch | 1,37 | .014 | .906 | < .001 |
| Treatment Order * Treatment * Trial Type * Switch | 1,37 | 2.857 | .099 | .072 |

The interaction further involving Trial Type indicated that the difference between reference and comparison trials was on average larger in the first session: for subjects receiving placebo first, the difference (reference minus comparison) was larger in the placebo than L-tyrosine condition (*M* = 113 vs 53 ms, *p* < .001), whereas for subjects receiving L-tyrosine first the difference was larger in the L-tyrosine than placebo condition (*M* = 116 vs 56 ms, *p* < .001). Conversely, the interaction involving Switch indicated that switch costs (switch minus repeat) were on average larger in the first session: for subjects receiving placebo first, the switch cost was larger in the placebo than L-tyrosine condition (*M* = 104 vs 51 ms, *p* = .002), whereas for subjects receiving L-tyrosine first the switch cost was larger in the L-tyrosine than placebo condition (*M* = 93 vs 49 ms, *p* < .001).

Importantly, it should be noted that because treatment order was counterbalanced across subjects, the order effect cannot account for the whole-sample variance in gate opening scores being smaller in the L-tyrosine than placebo condition, *t*(37) = 2.072, *p* = .023, as reported in the main text. The reduced variance in the L-tyrosine condition points to a reliable baseline-dependent effect of L-tyrosine that is not explainable either by regression to the mean or an order effect. Therefore, these analyses taken together indicate that L-tyrosine’s baseline-dependent effect on gate opening is reliable even when accounting for the order effect across sessions.

Subsequently, the abovementioned rmANOVAs were repeated but instead of Treatment Order they included Task Order (reference-back first vs Stroop first) or the version of the Raven performed during the waiting period (RPM vs APM) as between-subject factor, see Tables S2 and S3 respectively. In sum, these analyses revealed no significant interactions involving Treatment, thus indicating that these factors did not confound the baseline-dependent effect of L-tyrosine on gate opening.

| *Table S2.* Analysis of task order and reference-back performance (in RT) | | | | |
| --- | --- | --- | --- | --- |
| **Effect** | **DF** | ***F*** | ***p*** | ***η*^2^*_p_*** |
| Task Order | 1,37 | .192 | .663 | .005 |
| Treatment | 1,37 | .356 | .554 | .010 |
| Trial Type | 1,37 | 49.677 | **< .001** | .573 |
| Switch | 1,37 | 34.710 | **< .001** | .484 |
| Task Order * Treatment | 1,37 | .024 | .881 | < .001 |
| Task Order * Trial Type | 1,37 | .113 | .739 | .003 |
| Task Order * Switch | 1,37 | .001 | .971 | < .001 |
| Treatment * Trial Type | 1,37 | .756 | .390 | .020 |
| Treatment * Switch | 1,37 | .012 | .915 | < .001 |
| Trial Type * Switch | 1,37 | 2.36 | .133 | .060 |
| Task Order * Treatment * Trial Type | 1,37 | .748 | .393 | .020 |
| Task Order * Treatment * Switch | 1,37 | .006 | .941 | < .001 |
| Task Order * Trial Type * Switch | 1,37 | .408 | .527 | .011 |
| Treatment * Trial Type * Switch | 1,37 | .412 | .525 | .011 |
| Task Order * Treatment * Trial Type * Switch | 1,37 | .747 | .393 | .020 |

| *Table S3.* Analysis of Raven task type and reference-back performance (in RT) | | | | |
| --- | --- | --- | --- | --- |
| **Effect** | **DF** | ***F*** | ***p*** | ***η*^2^*_p_*** |
| Raven | 1,37 | .331 | .568 | .009 |
| Treatment | 1,37 | .538 | .468 | .014 |
| Trial Type | 1,37 | 122.764 | **< .001** | .768 |
| Switch | 1,37 | 86.114 | **< .001** | .699 |
| Raven * Treatment | 1,37 | .042 | .838 | .001 |
| Raven * Trial Type | 1,37 | .211 | .649 | .006 |
| Raven * Switch | 1,37 | 3.060 | .089 | .076 |
| Treatment * Trial Type | 1,37 | .139 | .711 | .004 |
| Treatment * Switch | 1,37 | .005 | .945 | < .001 |
| Trial Type * Switch | 1,37 | 2.525 | .121 | .064 |
| Raven * Treatment * Trial Type | 1,37 | 1.883 | .178 | .048 |
| Raven * Treatment * Switch | 1,37 | .159 | .693 | .004 |
| Raven * Trial Type * Switch | 1,37 | .020 | .887 | < .001 |
| Treatment * Trial Type * Switch | 1,37 | < .001 | .975 | < .001 |
| Raven * Treatment * Trial Type * Switch | 1,37 | .646 | .427 | .017 |

*Effect of L-tyrosine on gate opening is not driven by unusually high variance in the placebo condition*

Inspection of Figure 2C in the main text might raise the concern that the effect of L-tyrosine on gate opening performance is not due to a selective modulation of variability between subjects in the L-tyrosine condition but instead is driven by unusually high variability in the placebo condition. Indeed, variance in gate opening costs in the placebo condition is notably higher than variance in either condition for updating cost and gate closing cost (see Figure 2C). To address this concern, data was analyzed from an unpublished study in which 43 subjects completed an identical version of the reference-back task as used in the present study, but without any manipulation such as L-tyrosine administration. Mean behavioral cost in these unpublished data was similar as those observed in the present study’s placebo condition (Updating cost: 135 ms, Gate opening: 61 ms, Gate closing: 127 ms, compare with costs illustrated in Figure 2A in the main text). Critically, these unpublished data also revealed higher variance in gate opening (*S^2^* = 14992) as compared to updating cost (*S^2^* = 7704), *t*(41) = 2.247, *p* = .015, and gate closing (*S^2^* = 8671), *t*(41) = 1.921, *p* = .031, with the latter two not differing significantly from each other, *t*(41) = .393, *p* = .348. This indicates that, under normal circumstances, the variability between subjects in gate opening performance is higher than in updating and gate closing costs.

As such, the present study’s difference in gate opening variance between the L-tyrosine and placebo conditions was not driven by unusually high variance in the placebo condition. Instead, the results point to a selectively reduced variance in the L-tyrosine condition. This indicates a baseline-dependent effect of L-tyrosine on gate opening beyond regression to the mean.
